# Assessing the association between hypoxia during craniofacial development and oral clefts

**DOI:** 10.1590/1678-7757-2017-0234

**Published:** 2018-05-11

**Authors:** Erika Calvano Küchler, Lea Assed da Silva, Paulo Nelson-Filho, Ticiana M. Sabóia, Angela M. Rentschler, José Mauro Granjeiro, Driely Oliveira, Patricia N. Tannure, Raquel Assed da Silva, Leonardo Santos Antunes, Michael Tsang, Alexandre R. Vieira

**Affiliations:** 1 Universidade de São Paulo, Faculdade de Odontologia de Ribeirão Preto, Departamento de Odontopediatria, Ribeirão Preto, São Paulo, Brasil.; 2 Universidade Federal Fluminense, Unidade de Pesquisa Clínica, Niterói, Rio de Janeiro, Brasil.; 3 University of Pittsburgh School of Dental Medicine, Department of Oral Biology, Pittsburgh, PA, USA.; 4 Instituto Nacional de Metrologia, Qualidade e Tecnologia (INMETRO), Programa de Bioengenharia, Xerém, Rio de Janeiro, Brasil.; 5 Universidade Veiga de Almeida, Faculdade de Odontologia, Departamento de Odontopediatria, Rio de Janeiro, Rio de Janeiro, Brasil.; 6 Universidade Federal Fluminense, Faculdade de Odontologia de Nova Friburgo, Departamento de Formação Específica, Nova Friburgo, Rio de Janeiro, Brasil.; 7 University of Pittsburgh School of Medicine, Department of Developmental Biology, Pittsburgh, PA, USA.

**Keywords:** Gene, Genetic, Children

## Abstract

**Objectives:**

To evaluate the association between hypoxia during embryo development and oral clefts in an animal model, and to evaluate the association between polymorphisms in the HIF-1A gene with oral clefts in human families.

**Material and Methods:**

The study with the animal model used zebrafish embryos at 8 hours post-fertilization submitted to 30% and 50% hypoxia for 24 hours. At 5 days post-fertilization, the larvae were fixed. The cartilage structures were stained to evaluate craniofacial phenotypes. The family-based association study included 148 Brazilian nuclear families with oral clefts. The association between the genetic polymorphisms rs2301113 and rs2057482 in HIF-1A with oral clefts was tested. We used real time PCR genotyping approach. ANOVA with Tukey's post-test was used to compare means. The transmission/disequilibrium test was used to analyze the distortion of the inheritance of alleles from parents to their affected offspring.

**Results:**

For the hypoxic animal model, the anterior portion of the ethmoid plate presented a gap in the anterior edge, forming a cleft. The hypoxia level was associated with the severity of the phenotype (p<0.0001). For the families, there was no under-transmitted allele among the affected progeny (p>0.05).

**Conclusion:**

Hypoxia is involved in the oral cleft etiology, however, polymorphisms in HIF-1A are not associated with oral clefts in humans.

## Introduction

Oral clefts are a common birth defect seen worldwide^16^. Epidemiologically, oral clefts are separated in two distinct entities: cleft lip with or without cleft palate and cleft palate only. Oral clefts can also be divided into isolated (non-syndromic) and syndromic forms. Isolated oral clefts affect about 1.7 *per* 1,000 live births, with ethnic and geographic variation^15^. These conditions are complex alterations resulting from multiple genetic and environmental factors^16^.

Hypoxia is an environmental factor involved in the etiology of different birth defects^27^. Oral clefts are a possible hypoxia-induced birth defects. Some evidence in human populations suggests an association between these two conditions, such as maternal cigarette smoking, acardiac twining, and living in very high altitudes (>6500 feet from sea level)^6^ ^,^ ^7^ ^,^ ^10^ ^,^ ^13^. Data on birth defects in the highlands of South America showed a significantly higher risk for oral clefts when compared to the population living at sea level^6^.

Many studies assessed the association between maternal cigarette smoking during pregnancy and oral clefts. A meta-analysis of these studies suggested a positive association between oral clefts and maternal smoking^12^, as well as passive maternal smoking. Tobacco smoke contains thousands of compounds, some of which are known to have deleterious effects during embryonic development. Among smoke products, nicotine is considered to be the main teratogenic substance that alters and delays embryonic development^21^. The carbon monoxide in tobacco smoke is another mechanism that may cause oral clefts due to maternal smoking, since it reduces the availability of oxygen to the fetus, causing hypoxia ^9^ ^,^ ^13^.

Embryonic development occurs under a certain degree of hypoxia, in which the expression of Hypoxia-Inducible Factors (HIFs) is critical. HIFs are transcription factors that respond to changes in available oxygen in the cellular environment. HIF-1A (Hypoxia-inducible factor 1-alpha) activates the transcription of genes that are involved in crucial aspects of development, including angiogenesis and cell survival^27^. HIF-1A is expressed in most, if not all, human tissues. HIF-1A seems to play a very general role by signaling the existence of hypoxia to the transcriptional machinery in the nucleus of all cells^23^. An *in vitro* study with hypoxic embryos suggested the role of HIF-1A in oral cleft etiology^17^.

This study had two main objectives: 1) to evaluate the influence of the hypoxia condition in the craniofacial formation; and 2) to evaluate the association between oral cleft in humans and HIF-1A. Therefore, we used zebrafish as a model to evaluate the association between hypoxia during embryo development and oral clefts; and we evaluated the association between genetic polymorphisms in HIF-1A with oral clefts in human families.

## Material and methods

### Animal model and hypoxic conditions

All procedures involving zebrafish (*Danio rerio*) were performed with the prior review and approval from the University of Pittsburgh Institutional Animal Care and Use Committee (Protocol #: 12020279). Fertilized eggs were obtained from zebrafish according to the procedures described by Westfield^27^ (1995). The eggs were collected within 1-hour post-fertilization (hpf), pooled, and placed into E3 (5 mM NaCl, 0.17 mM KCl, 0.33 mM CaCl_2_, 0.33 mM MgSO_4_).

We performed a pilot study to establish a protocol that allows the study of the association between hypoxic conditions during embryonic development and clefts in zebrafish. Sample size was also estimated according to the results observed in the pilot study. We repeated the experimental condition described above three times with 10 embryos in each group to assure that the results were consistent.

The hypoxia experiment was performed with two experimental hypoxic condition groups: 30% hypoxic condition and 50% hypoxic condition. We created a control group for comparison. We used 105 samples (35 *per* group).

Embryos at 8 hpf (right before neural crest cell migration) the embryos were submitted to 30% and 50% hypoxia treatment until 32 hpf developmental stage (stage where the face structures are forming). After the hypoxia treatment, the embryos were transferred to a normoxic condition until reaching 5 days post-fertilization (dpf).

For the hypoxia treatment, the oxygen level was reduced by bubbling nitrogen gas into the water. The oxygen levels were monitored using a dissolved oxygen meter (SonTek/YSI model 58, Fisher Scientific, Pittsburgh, PA, USA). Zebrafish eggs were transferred to one of the incubation regimes (30% or 50% hypoxia). The containers (50 mL Falcon tube, Falcon Labware, Oxnard, CA, USA) with the treated water were sealed.

At 5 dpf, the zebrafish larvae were fixed with paraformaldehyde. The cartilage structures were stained with Alcian Blue solution to evaluate craniofacial phenotype in the lower and upper jaws^26^.

**Figure 1:**
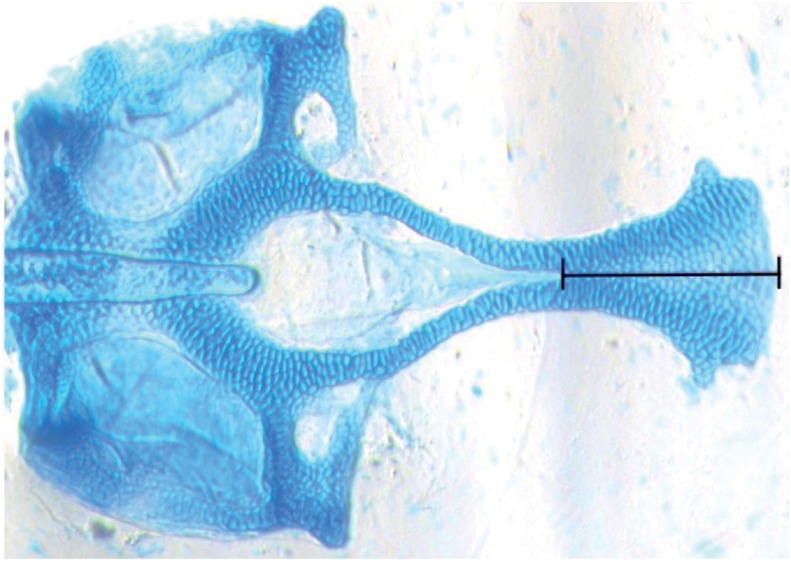
Ventral view. Landmarks used to measure the length of the anterior portion of the ethmoid plate formation

We obtained the images using a Leica M165 stereomicroscope and processed them in Adobe Photoshop CS. Two landmarks were collected on ventral image specimens for a measurement analysis. Figure 1 shows both landmarks located on the ethmoid plate (upper jaw). The distance between these landmarks was measured in millimeters using ImageJ software to establish how much of the ethmoid plate formed. Lower values mean less ethmoid plate formation and deeper clefts. The only images used for analyses were those with high quality and integrity of the zebrafish larvae.

### Family-based association study

This study was approved by the Human Ethics Committee of the Health Department of the city of Rio de Janeiro (#113/09). All participating individuals and parents signed Informed Consent Forms. We included 148 nuclear families treated at the Nossa Senhora do Loreto Hospital, Rio de Janeiro, Brazil. Syndromic oral cleft cases were excluded; the characteristics of these families were previously described^22^.

The cleft phenotype was determined based on the description in the clinical records, which included type of cleft (cleft lip only with or without cleft palate, and cleft palate only). The ethnicity definition was based on self-reported information. The positive family history of oral cleft was also based on self-reported information related to first, second and third degree-relatives of the proband. Data regarding hypoxia (smoking habits and area of residence during the first trimester, and twinning or higher multiple births) were obtained through standardized questionnaires.

We collected saliva samples for DNA extraction. The genomic DNA was extracted from buccal cells based on the previously published protocol^11^. The selected genetic polymorphisms were rs2301113 and rs2057482 in HIF-1A; located in 14q23.2. The samples were genotyped by real-time polymerase chain reactions using the TaqMan assay^20^ in a StepOnePlus real-time PCR instrument (Applied Biosystems, Foster City, CA, USA).

### Statistical analyses

We used Graph Pad Prism 5.0a (Software, La Jolla, CA, USA) to analyze data from the animal model. The Shapiro-Wilk test was used to verify the normality of the data. One-way ANOVA with Tukey's post-test was used to compare the means of the ethmoid plate lengths. We made comparisons between all possible groups.

We used the Plink software (Version 1.06, http://pngu.mgh.harvard.edu/purcell/plink/) to analyze the polymorphism associations. The transmission/disequilibrium test (TDT) was used to check the distortion of the inheritance of alleles from heterozygous parents to their affected offspring^24^, and thus to test for association between each HIF-1A polymorphism and a susceptibility for oral clefts subgroups. We used an established alpha of 5%.

## Results

### Animal model

**Figure 2:**
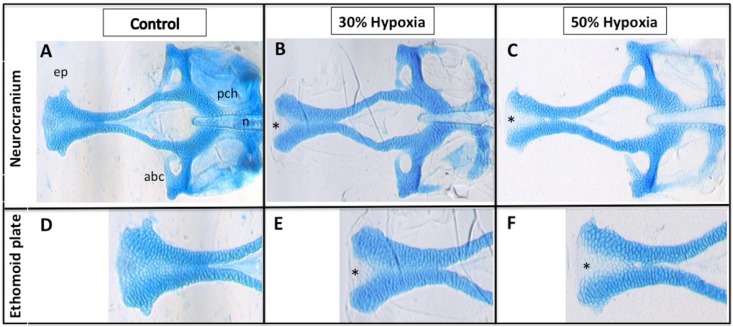
Hypoxia results in craniofacial defects. (A-F) Ventral view of 5 dpf larvae stained with Alcian Blue; (A-C) Dissection of the neurocranium; (D-F) Closer view of the ethmoid plate; (B, C, E and F) Morphological alterations in the anterior area of the ethmoid plate, including a gap in the anterior edge forming a cleft; (C and F) Results of a slightly more severe phenotype in the ethmoid plate (deeper cleft). abc= anterior basicapsular commissure; ep= ethmoid plate; n= notochord; pch= parachordal; * indicates cleft in the ethmoid plate

We observed no differences between the incubation regime and mortality (*p*>0.05). The results showed that hypoxia resulted in obvious craniofacial malformations regardless of the hypoxia level. No groups presented phenotype alteration in the lower jaw. However, we observed a tissue hypoplasia in the anterior portion of the ethmoid plate in the upper jaw, including a gap in the anterior edge, forming a cleft. Figure 2 shows the phenotype alterations observed in the ethmoid plate in both hypoxic conditions in comparison to the control group.

**Figure 3:**
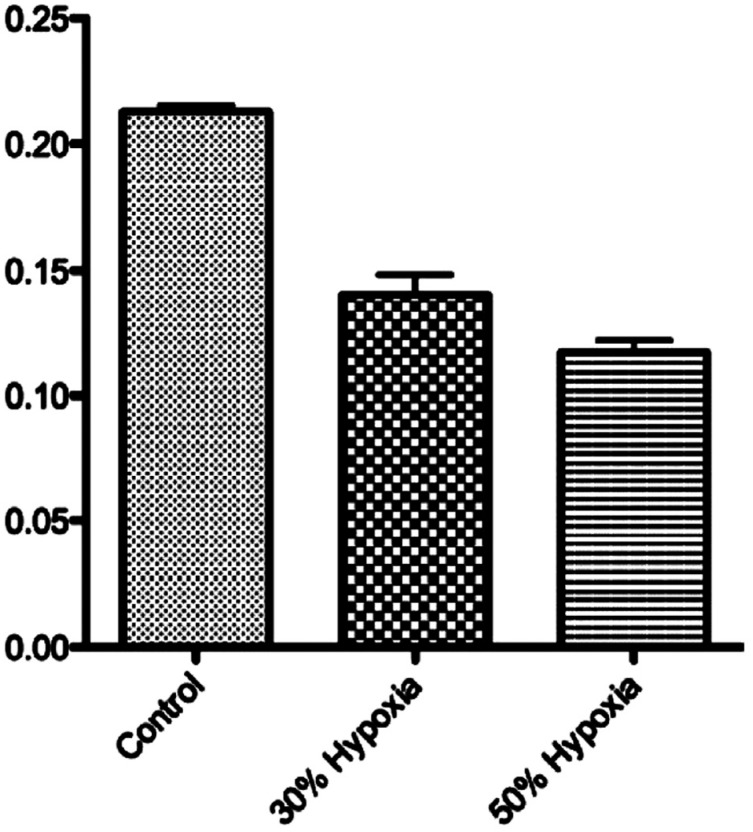
Length of the anterior portion of the ethmoid plate in wild larvae and the two different hypoxia groups. Differences were observed between all groups p<0.0001

The analysis of ethmoid plate formation showed statistical difference between the groups (Figure 3). Differences were observed between all groups (*p*<0.0001).

### Family-based study

We included 530 subjects in this study (148 oral cleft nuclear families). In 78 families, both parents were included in the study (complete triads); in 61 families, only the mother was available, and in 9 families, only the father was available (70 dyads). One hundred twenty five had cleft lip with or without cleft palate (27 cleft lip only and 98 cleft lip and palate) and 23 had cleft palate only.

Regarding the area of residence, all mothers reported to live at the sea level during the first trimester of pregnancy. None of the cases were twins or higher multiple births. Twenty (13.5%) mothers reported smoking during the first trimester of pregnancy. Table 1 presents the characteristics of the sample.

**Table 1:** Characteristics of the included families

**Age, years (SD)**	
Probands	8.14 (5.7)
Mothers	35.29 (8.5)
Fathers	41.39 (27.8)
**Probands gender (%)**	
Male	86 (58.1)
Female	62 (41.9)
**Probands ethnic background (%)**	
Caucasian	71 (48.0)
African descent	77 (52.0)
Maternal smoking during the first trimester(%)	20 (13.5)
Positive family history of oral cleft (%)	64 (43.2)

Note: SD: standard deviation

Table 2 presents the TDT results. There was no under-transmitted allele among the affected progeny. Furthermore, when the analysis was performed in the subgroup with history of maternal smoking during the first trimester of their pregnancy, or positive history of oral cleft, there was no association between the studied polymorphisms in HIF-1A and oral clefts (*p*>0.05).

**Table 2:** Association between oral cleft subgroups and HIF-1A polymorphisms

Polymorphism	Subgroups	Number of informative families	Allele of reference	T	U	p-value
rs2301113	All clefts Cleft lip with or without cleft palate Cleft palate	66 46 19	A	35 25 10	31 21 9	0.622 0.555 0.818
rs2057482	All clefts Cleft lip with or without cleft palate Cleft palate	60 44 16	C	30 19 11	30 25 5	1 0.36 0.2

Note: T: Transmitted and U: Untransmitted

## Discussion

Some experimental *in vivo* studies^4^ ^,^ ^14^ ^,^ ^17^ and observational studies^6^ ^,^ ^9^ ^,^ ^10^ ^,^ ^13^ suggest that hypoxia in the first trimester can increase the risk of oral clefts. To the best of our knowledge, our study is the first to observe that hypoxia during embryonic development can cause oral clefts in zebrafish. Zebrafish are an important model in the study of developmental biology and organogenesis^1^ ^,^ ^3^ ^,^ ^7^ ^,^ ^18^. For this reason, we decided to use zebrafish embryos as a model to evaluate the effect of severe levels of hypoxia on the palate development.

We were able to note that hypoxia caused clefts in all treated larvae. In addition, we observed that the level of hypoxia was directly related to the severity of the cleft. In the experimental group with 50% hypoxia, there were deeper clefts than in the group with 30% hypoxia. Zebrafish are a valuable model to study jaw development due to having the same skeletal elements as higher vertebrates^25^, however, it is important to consider the extent to which mechanisms for palatogenesis are conserved across the species. The mammalian palate development is a process in which primary and secondary palatal shelves develop as outgrowths from the medial nasal and maxillary prominences, respectively, remodel, and fuse to form the intact roof of the oral cavity^8^. The zebrafish palate development consists of a series of bones in the roof of the mouth that separate the oral cavity from the brain. The development of this structure does not involve palatal shelf formation, but instead the condensation of cranial neural crest-derived mesenchyme^5^.

Some studies propose that oral clefts could result from interference with neural crest cell migration^2^. In our study, we performed the hypoxia treatment before the migration of cranial neural crest cells. In zebrafish, the migratory cranial neural crest cells arise from dorsal and lateral regions of the neural ectoderm and are identifiable at 12 hpf^7^. The hypoxic condition could be related to reduced numbers of cranial neural crest cells in the pharyngeal arches or that hypoxia alters the proliferation or migration of cranial neural crest cells.

In a previous study performed with a whole embryo culture system, hypoxic embryos presented a high frequency of impaired fusion. In these embryos, cell proliferation activity was decreased in the fusion areas following downregulation of genes that are involved in lip formation. These results suggest that embryonic hypoxia during lip and palate formation induces apoptosis in physiologically hypoxic regions and HIF expression^17^. Therefore, we decided to evaluate if polymorphisms in HIF-1A are involved in the etiology of oral clefts in Brazilian families.

HIF-1 is considered a master switch that allows cells to respond to falling oxygen levels. Many genes are transcriptionally activated by HIF-1A in response to hypoxia, including important genes for the correct lip and palate formation. Our results showed that the polymorphisms rs2301113 and rs2057482 in HIF-1A are not associated to oral clefts etiology.

Authors of a study performed with 2524 mothers who gave birth to babies with an oral cleft, hypothesize that the severity of oral cleft might increase due to hypoxia resulting from gestational bleeding^19^. Our study, with an animal model, suggested that higher levels of hypoxia are associated with a more severe cleft phenotype. However, our familial study did not observe any preferential under-transmitted allele according to the cleft type.

We examined a gene-environment interaction to verify if polymorphisms in HIF-1A are associated with oral clefts in a subgroup that had history of maternal smoking during pregnancy, which is a condition that causes oral clefts due to the reduced oxygen. In fact, animal studies show that the administration of nicotine during pregnancy decreases uterine blood flow^29^. We did not find an association. However, the sample size of those who reported maternal smoking during pregnancy might be too small for this type of analysis. Polymorphisms in genes that express nicotinic receptors could be involved in oral clefts, since studies report the effects nicotinic receptors on the uterine arteries^29^.

Despite the association between hypoxia and oral cleft being reported in literature, genes and their function in neural crest induction, specification and differentiation started recently. The genetic circuits that coordinate this complex developmental process in humans is still largely unknown. Our data from the family analysis should be interpreted with caution. Although our results did not show an association between polymorphisms in HIF-1A and oral clefts, we can hypothesize that other polymorphisms in HIF-1A are associated with oral clefts. Since all mothers lived at sea level during pregnancy, this could be the reason we were not able to detect any association.

## Conclusion

Our results support the hypothesis that hypoxia plays an important role in the oral cleft etiology. However, we were not able to find an association between polymorphisms in HIF-1A and oral clefts in humans.
